# Supplementary material for: Cerebrospinal fluid proteomics for predictive assessment of Alzheimer’s Disease risk
Source: medRxiv. 2025 Oct 27:2025.10.24.25337921. Preprint. [Version 1] doi: 10.1101/2025.10.24.25337921 (PMC12636643; doi:10.1101/2025.10.24.25337921)
Supplement: 1 [file NIHPP2025.10.24.25337921V1-supplement-1.pdf]

medRxiv preprint doi: https://doi.org/10.1101/2025.10.24.25337921; this version posted October 27, 2025. The copyright holder for this preprint (which was not certified by peer review) is the author/funder, who has granted medRxiv a license to display the preprint in perpetuity.
It is made available under a CC-BY 4.0 International license .

Supplementary Figures:

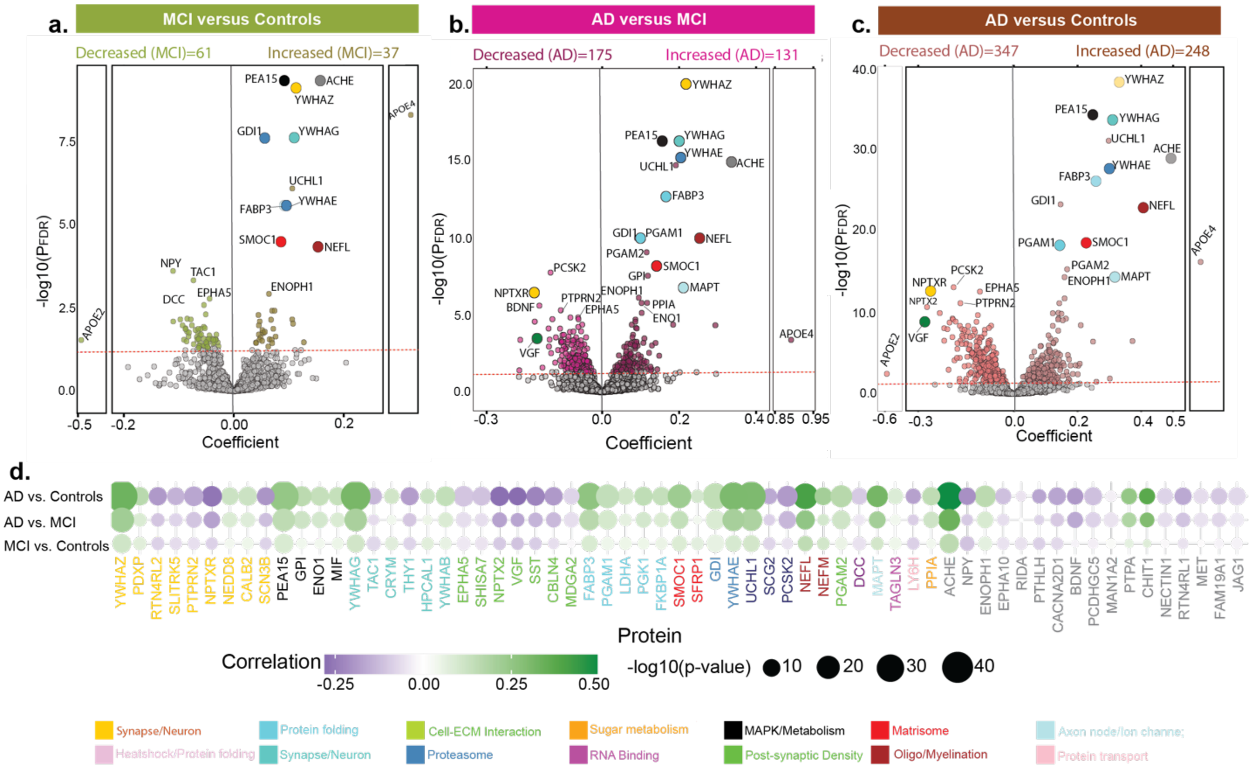

Fig. S1. Differential abundance of CSF proteins across clinically diagnosed groups in ADNI. *a-c,* Volcano plots showing differential proteins abundance between ***a,*** MCI versus Controls (Increased: 37, Decreased: 61), ***b,*** AD versus MCI (Increased: 131, Decreased: 175)**,** and ***c,*** AD versus Controls (Increased: 248, Decreased: 347) (Controls: n=377, MCI: n=563, AD: n=164). The dashed red line indicates the significance threshold at α = 0.05 after FDR correction. Top proteins are labeled for clarity, and selected proteins of interest are zoomed in. The x axis represents the regression coefficient while the y axis shows the -log10 of the FDR-corrected p-value calculated for each protein. ***d,*** Heatmap of regression coefficients showing associations between CSF proteomic profiles and clinical diagnosis. CSF proteins are labeled by their respective gene symbols, and the strength and direction of correlation are indicated by the purple-to-green color scale. The top 30 proteins were selected (based on P value) from each category, and their union is displayed in the heatmaps. Colors are assigned to individual protein names based on their membership in the brain-derived modules[17], consistent across both the volcano plots and heatmaps. In the heatmap, proteins shown in gray were either not present in the prior network study or were not assigned to any module[17].

medRxiv preprint doi: https://doi.org/10.1101/2025.10.24.25337921; this version posted October 27, 2025. The copyright holder for this preprint (which was not certified by peer review) is the author/funder, who has granted medRxiv a license to display the preprint in perpetuity.
It is made available under a CC-BY 4.0 International license .

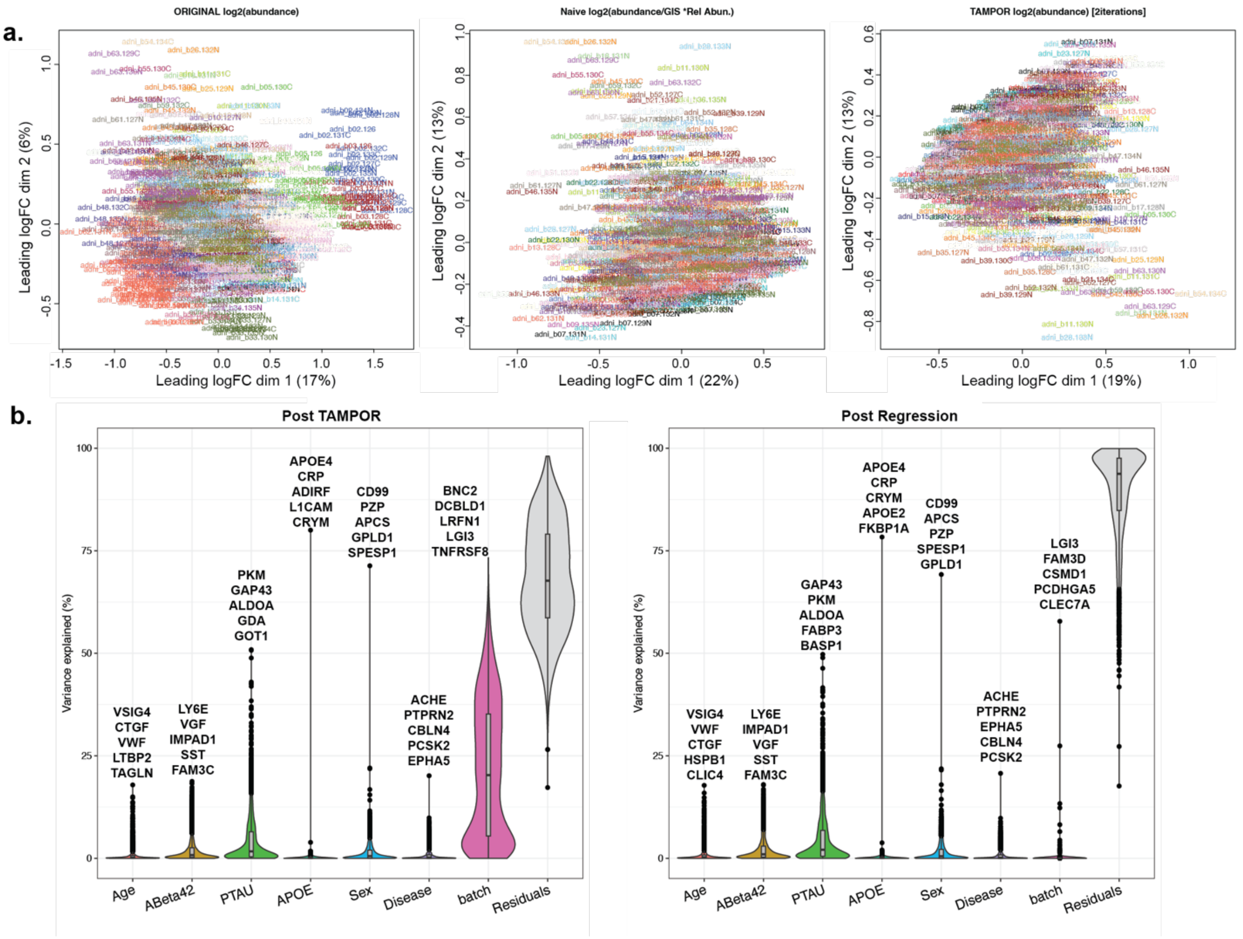

Fig. S2. Quality control of the ADNI CSF proteome. a, Multidimensional scaling (MDS) illustrating TMT-MS batch correction. Log_2 abundance, log_2 abundance divided by the global internal standard (GIS), and TAMPOR are shown. **b,** Variance partition plots were used to visualize the percent variance of each protein in the dataset co-varying with batch, age and sex. The matrix was subjected to bootstrap regression (right) to remove variance due to batch.

medRxiv preprint doi: https://doi.org/10.1101/2025.10.24.25337921; this version posted October 27, 2025. The copyright holder for this preprint (which was not certified by peer review) is the author/funder, who has granted medRxiv a license to display the preprint in perpetuity.
It is made available under a CC-BY 4.0 International license .

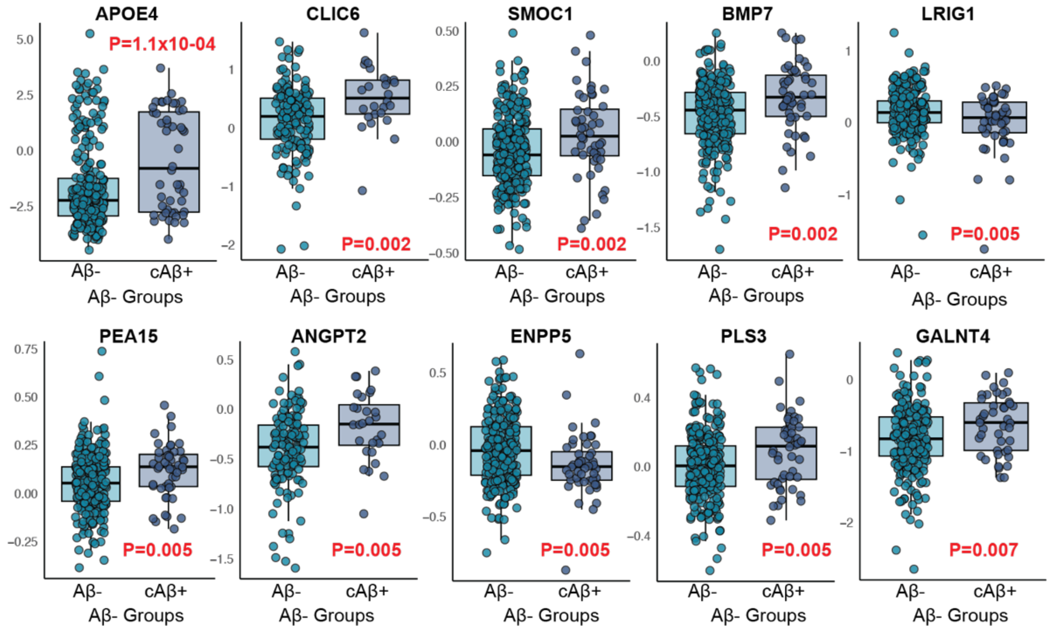

Fig. S3. Proteins significantly associated with pathological onset. Box plots show abundance of top 10 proteins identified by differential abundance analysis between Aβ- participants who remained stable (Aβ-) and those who converted to Aβ+ (cAβ+). P values for each protein were obtained using a linear regression model. Box plots display the median and interquartile range (25th-75th percentile), with whiskers extending up to 1.5 times the interquartile range. Data points represent individual participants.

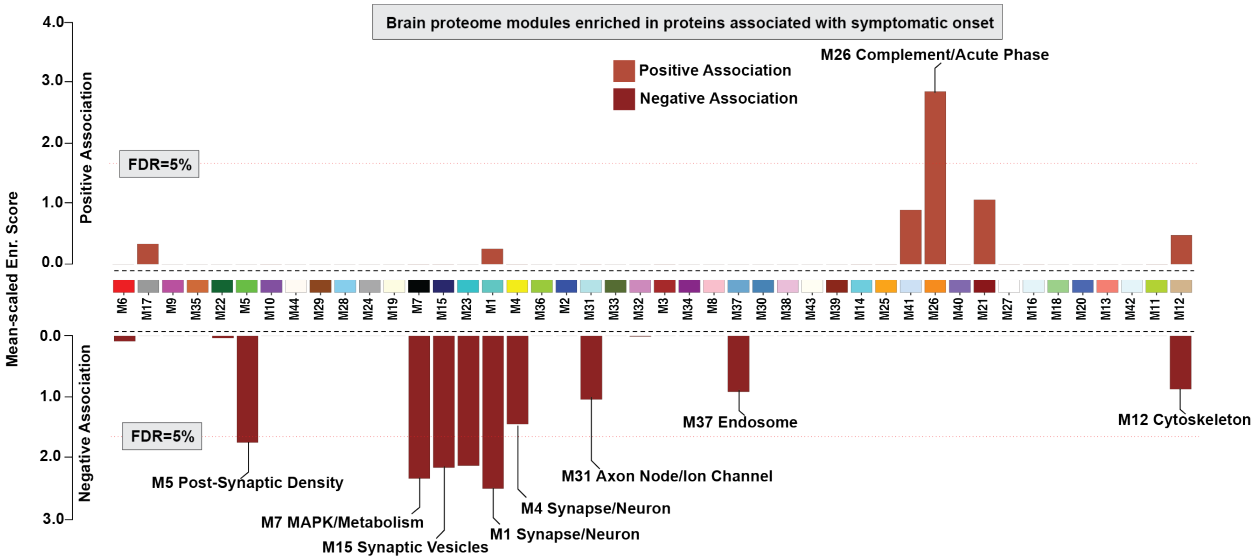

Fig. S4. Brain network modules (M5, M7, M15, M1, and M26) enriched for CSF biomarkers of symptomatic onset. CSF proteins associated with symptomatic onset (P<0.05) were mapped onto brain proteome network modules [17]. The horizontal red dotted line marks the 5% false discovery rate (FDR) threshold from permutation testing, above which the enrichment was considered significant. Modules with significant enrichment or biologically relevant are labeled, with proteins positively and negatively associated shown separately to highlight directionality. Unlabeled but significant modules lacked defined annotations in the original network study.

medRxiv preprint doi: https://doi.org/10.1101/2025.10.24.25337921; this version posted October 27, 2025. The copyright holder for this preprint (which was not certified by peer review) is the author/funder, who has granted medRxiv a license to display the preprint in perpetuity.
It is made available under a CC-BY 4.0 International license .

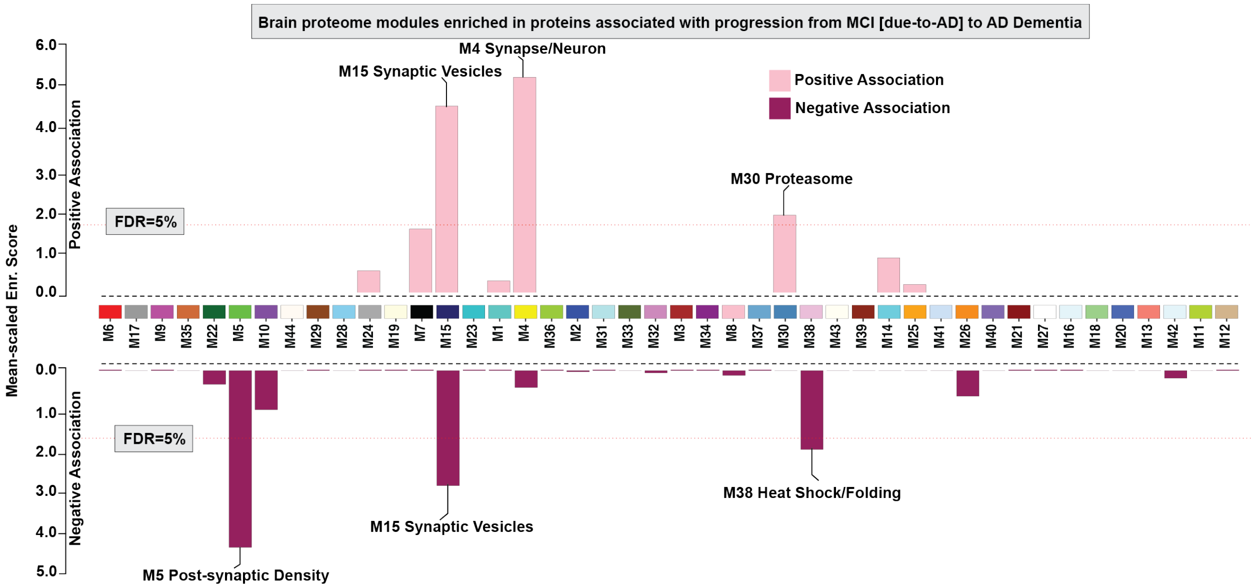

Fig. S5. Brain network modules (M5, M15, M4, M30, and M38) enriched for CSF biomarkers of disease progression from MCI [due-to-AD] to AD Dementia. CSF proteins associated with disease progression (P<0.05) were mapped onto brain proteome network modules[17]. The horizontal red dotted line marks the 5% false discovery rate (FDR) threshold from permutation testing, above which the enrichment was considered significant. Modules with significant enrichment are labeled, with proteins positively and negatively associated shown separately to highlight directionality. Some proteins negatively and others positively associated with progression were enriched in module M15.

medRxiv preprint doi: https://doi.org/10.1101/2025.10.24.25337921; this version posted October 27, 2025. The copyright holder for this preprint
(which was not certified by peer review) is the author/funder, who has granted medRxiv a license to display the preprint in perpetuity.
It is made available under a CC-BY 4.0 International license .

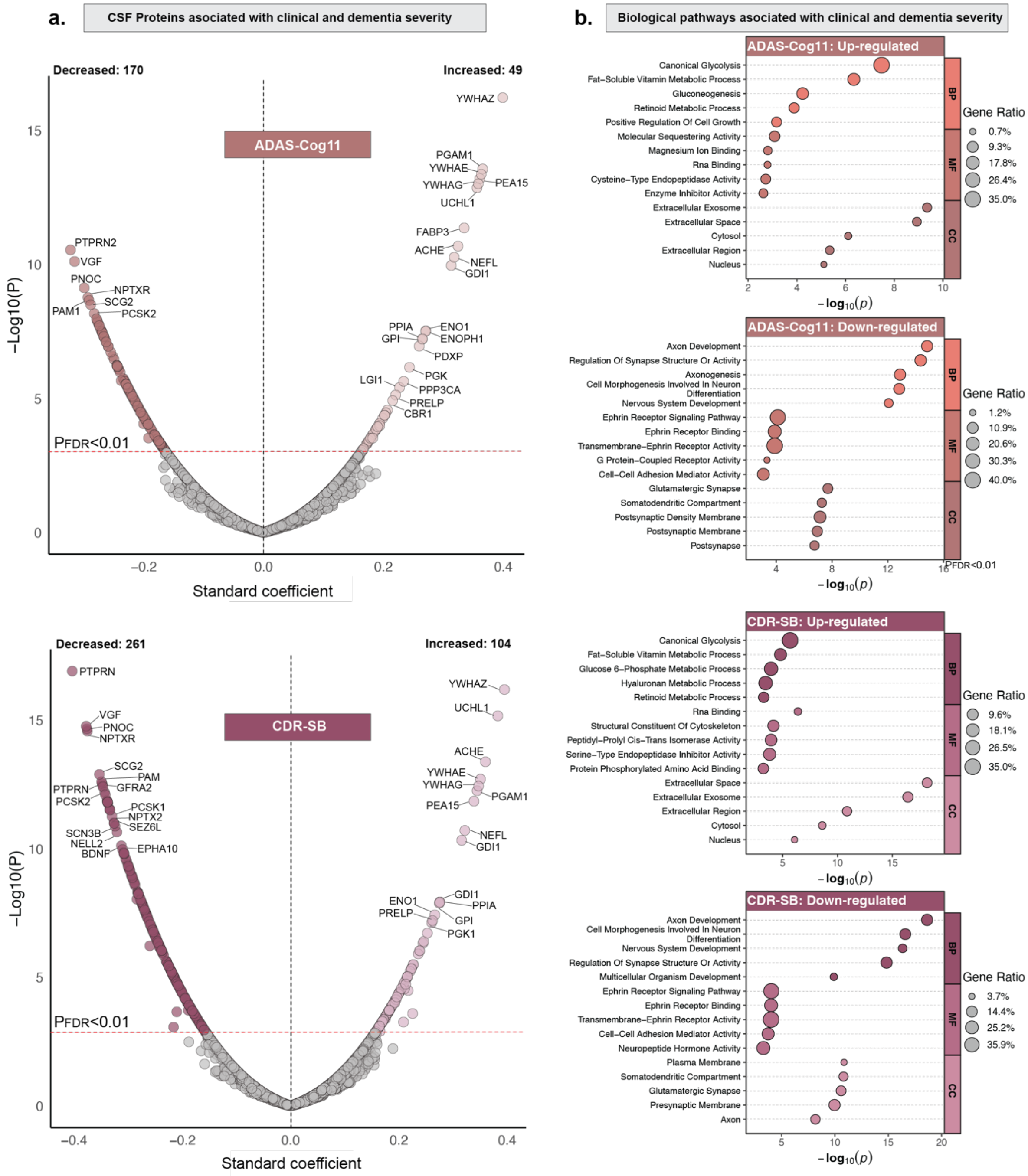

Fig. S6. Biological processes underlying the rate of change in clinical and dementia severity measures. *a,* Volcano plots depict differentially expressed proteins associated with the rate of change in cognitive (ADAS-Cog11; Increased: 49; Decreased: 170) and dementia severity measures (CDR-SB; Increased: 104; Decreased: 261). Linear mixed-effects models were used to estimate subject-specific rates of change separately for all outcomes. P values were adjusted for FDR, and the red line represents the threshold of $P_{FDR}<0.01$, above which proteins were considered significant. The x-axis shows the regression coefficient, and the y-axis indicates the -log10(P) for all proteins. Only the top proteins are labeled for legibility on the volcano plots. ***b,*** Gene ontology analysis was performed to identify biological processes associated with the rate of change in cognitive (ADAS-Cog11) and dementia severity measures (CDR-SB) using proteins significantly associated with each clinical measure at P<0.05, as shown in Panels **(a)** and **Fig. 5**. Upregulated and downregulated pathways are separately shown to highlight the directionality of the associations. Sample sizes were: ADAS-Cog11 (N = 406), and CDR-SB (N = 414).

medRxiv preprint doi: https://doi.org/10.1101/2025.10.24.25337921; this version posted October 27, 2025. The copyright holder for this preprint (which was not certified by peer review) is the author/funder, who has granted medRxiv a license to display the preprint in perpetuity.
It is made available under a CC-BY 4.0 International license .

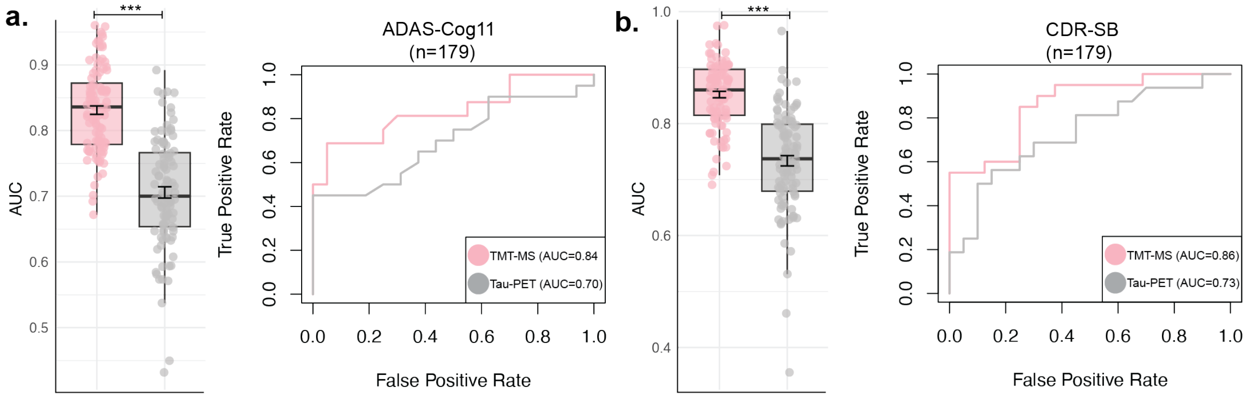

Fig. S7. CSF proteomics predicts longitudinal trajectories of clinical and dementia severity measures, beyond tau-PET imaging biomarker. Longitudinal trajectories of participants in terms of cognitive (ADAS-Cog11) and dementia severity (CDR-SB) measures were calculated leveraging participants having at-least 3 timepoints and data available for at least 24 months. Participants were divided into fast- versus stable/slow progressors based on median cutoff slope. Bar plot show the AUC of the models across 100 permuted runs in predicting stable/slow- and fast- progressors in longitudinal disease trajectories and the ROC curve showing the classification performance pertaining to median AUC is shown for **a.** ADAS-Cog11 and **b.** CDR-SB, using the following predictors: 1) the CSF proteins ("TMT-MS"), and 2) regional free-surfer based tau-PET SUVR. (*$p < 0.05$, **$p < 0.01$, ***$p < 0.001$). Sample sizes are: ADAS-Cog11: n=179 and CDR-SB: n = 179.

medRxiv preprint doi: https://doi.org/10.1101/2025.10.24.25337921; this version posted October 27, 2025. The copyright holder for this preprint (which was not certified by peer review) is the author/funder, who has granted medRxiv a license to display the preprint in perpetuity.
It is made available under a CC-BY 4.0 International license .

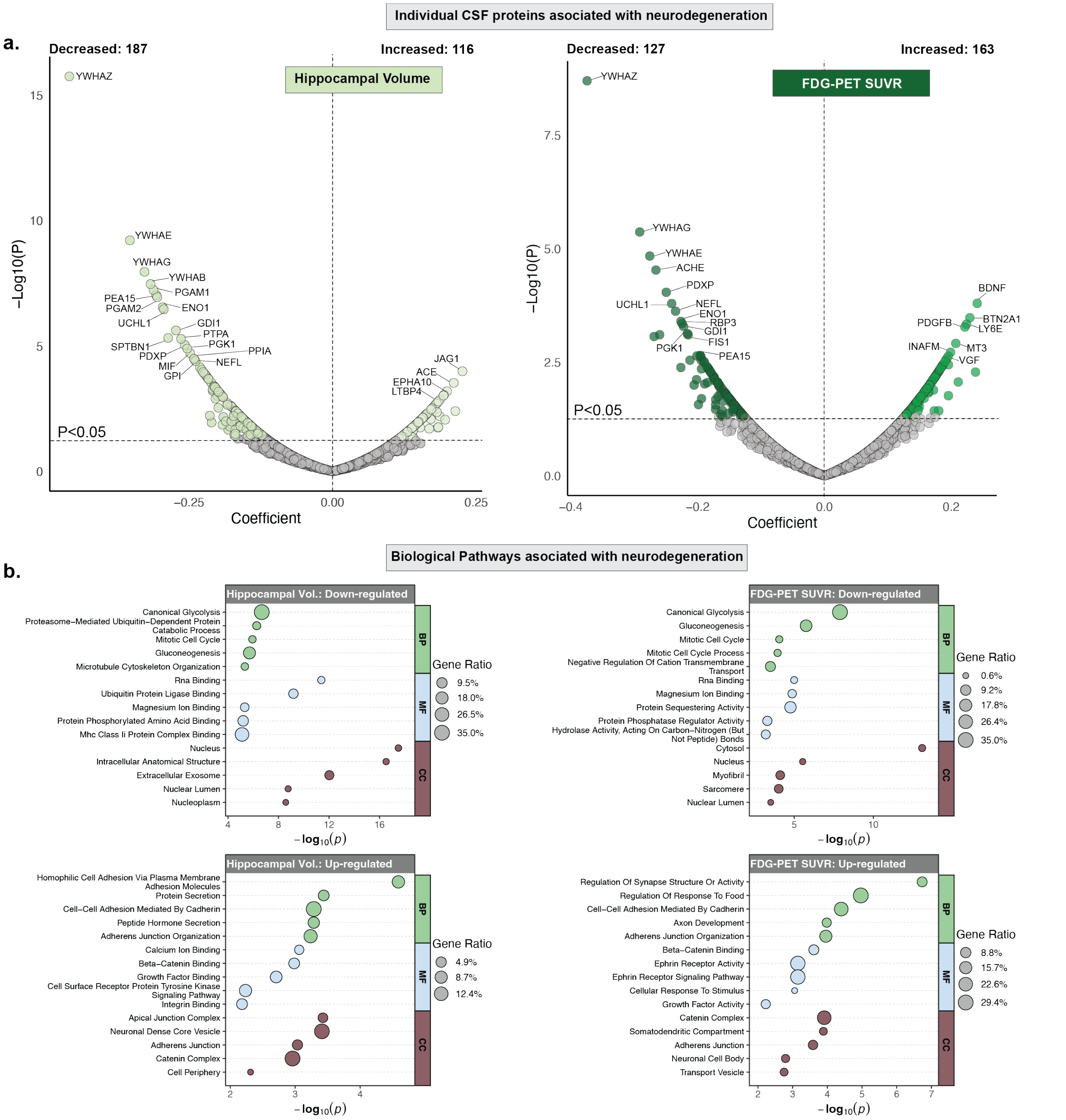

Fig. S8. Biological processes underlying the rate of change in neuroimaging-based neurodegeneration. ***a,*** Volcano plots depict differentially abundant proteins associated with the rate of change in neurodegeneration-related measures, including hippocampal volume, normalized by intracranial volume (Increased: 116; Decreased: 187) and FDG-PET SUVR, defined as the mean of angular, temporal, and posterior cingulate regions (Increased: 163; Decreased: 127). Linear mixed-effects models were used to estimate subject-specific rates of change separately for all outcomes. The black line indicates the threshold of P<0.05, above which proteins were considered significant. The x-axis shows the regression coefficient, and the y-axis indicates the -log10(P) for all proteins. Only the top proteins are labeled for legibility on the volcano plots. ***b,*** Gene ontology analysis was performed to identify biological processes associated with the rate of change in hippocampal volume and FDG-PET SUVR using proteins significantly associated with each imaging biomarker, as shown in Panels ***(a)*** and **Fig. 6**. Upregulated and downregulated pathways are shown separately to highlight the directionality of associations. Sample sizes were: Hippocampal volume (N = 290) and FDG-PET SUVR (N = 241).

medRxiv preprint doi: https://doi.org/10.1101/2025.10.24.25337921; this version posted October 27, 2025. The copyright holder for this preprint
(which was not certified by peer review) is the author/funder, who has granted medRxiv a license to display the preprint in perpetuity.
It is made available under a CC-BY 4.0 International license .

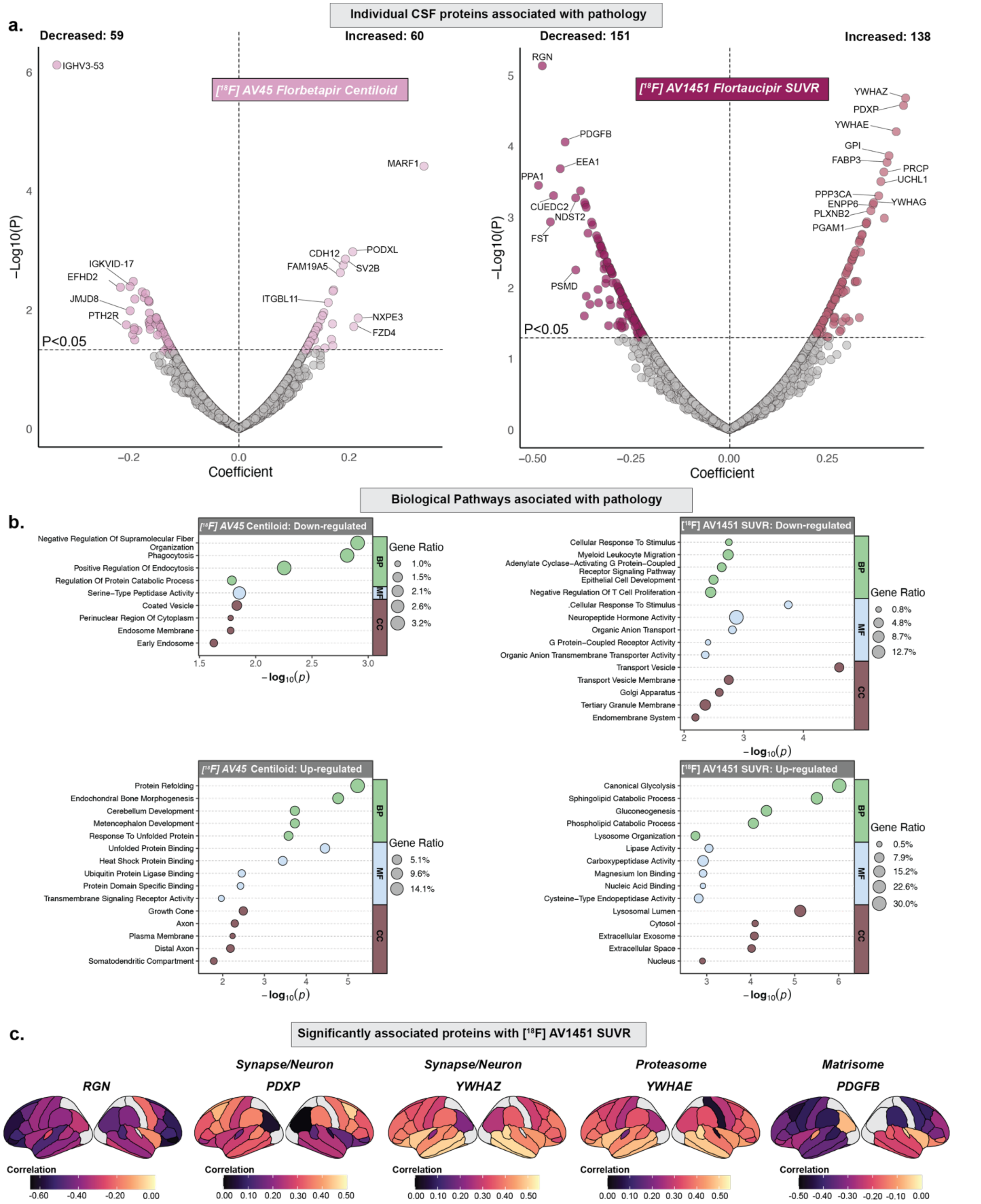

Fig. S9. Biological processes underlying the rate of change in neuroimaging-based pathological accumulation. ***a,*** Volcano plots depict differentially expressed proteins associated with the rate of change in amyloid accumulation ([^{18}F] AV45 PET Centiloid scale; Increased: 60; Decreased: 59) and neocortical tau accumulation ([^{18}F] AV1451 PET SUVR; Increased: 138; Decreased: 151). Linear mixed-effects models were used to estimate subject-specific rates of change separately for all outcomes. The black line indicates the threshold of P<0.05, above which proteins were considered significant. The x-axis shows the regression

medRxiv preprint doi: https://doi.org/10.1101/2025.10.24.25337921; this version posted October 27, 2025. The copyright holder for this preprint (which was not certified by peer review) is the author/funder, who has granted medRxiv a license to display the preprint in perpetuity.
It is made available under a CC-BY 4.0 International license .

coefficient, and the y-axis indicates the -log10(P) for all proteins. Only the top proteins are labeled for legibility on the volcano plots. ***b,*** Gene ontology analysis was performed to identify biological processes associated with the rate of change in amyloid (([^{18}F] AV45 PET Centiloids scale) and tau accumulation ([^{18}F] AV1451 PET SUVR) using the proteins significantly associated with each imaging phenotype, as shown in panel **a** and **Fig. 6.** Upregulated and downregulated pathways are separately shown to highlight the directionality of the associations. ***c,*** Surface maps show correlations between regional tau-PET SUVR accumulation and top-most selected five proteins from panel **a**. Sample sizes were: Aβ-PET Centiloids (N = 270), and [^{18}F] AV1451 PET SUVR (N = 83).

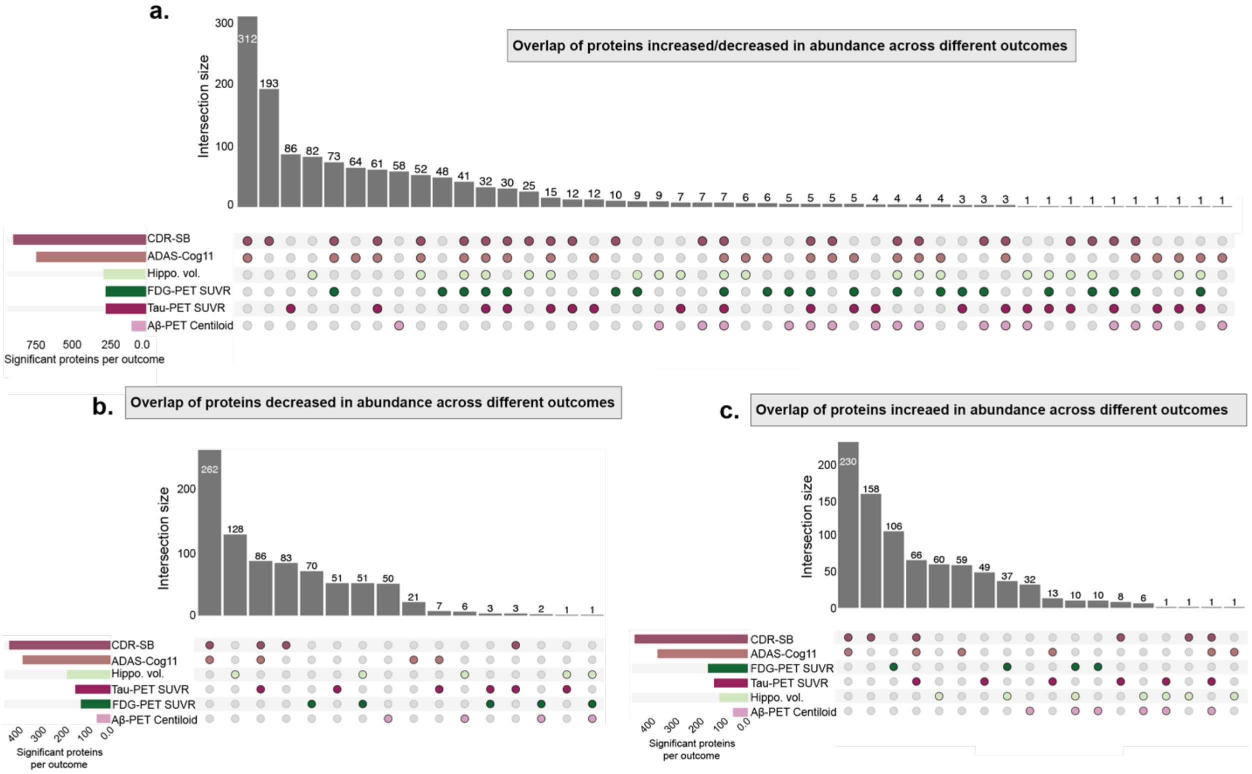

Fig. S10. UpSet plots showing the overlap of CSF proteins significantly associated with cognitive and dementia severity outcomes, as well as with neurodegeneration- and pathology-related measures. ***a,*** All significant proteins (increased or decreased in abundance); ***b,*** Proteins showing decreased abundance. ***c,*** Proteins showing increased abundance. Only FDR-significant proteins (P < 0.05) were included in the overlap analysis.
